# Correlation Analysis between Polysomnography Diagnostic Indices and Heart Rate Variability Parameters among Patients with Obstructive Sleep Apnea Hypopnea Syndrome

**DOI:** 10.1371/journal.pone.0156628

**Published:** 2016-06-02

**Authors:** Xuehao Gong, Leidan Huang, Xin Liu, Chunyue Li, Xuhua Mao, Weizong Liu, Xian Huang, Haiting Chu, Yumei Wang, Wanqing Wu, Jun Lu

**Affiliations:** 1 Department of Ultrasound, First Affiliated Hospital of Shenzhen University, Second People’s Hospital of Shenzhen, Shenzhen, Guangdong, China; 2 Guangzhou Medical University, Guangzhou, Guangdong, China; 3 Institute of Biomedical and Health Engineering, Shenzhen Institutes of Advanced Technology, Chinese Academy of Sciences, Shenzhen, Guangdong, China; 4 Department of Thyroid and Breast Surgery, First Affiliated Hospital of Shenzhen University, Second People’s Hospital of Shenzhen, Shenzhen, Guangdong, China; 5 Anhui Medical University, Hefei, Anhui, China; 6 Department of Respiratory Medicine, Xili People’s Hospital of Shenzhen, Shenzhen, Guangdong, China; 7 Department of Ultrasound, Second Clinical College of Jinan University, People’s Hospital of Shenzhen, Shenzhen, Guangdong, China; University of Rome Tor Vergata, ITALY

## Abstract

Heart rate variability (HRV) can reflect the changes in the autonomic nervous system (ANS) that are affected by apnea or hypopnea events among patients with obstructive sleep apnea hypopnea syndrome (OSAHS). To evaluate the possibility of using HRV to screen for OSAHS, we investigated the relationship between HRV and polysomnography (PSG) diagnostic indices using electrocardiography (ECG) and PSG data from 25 patients with OSAHS and 27 healthy participants. We evaluated the relationship between various PSG diagnostic indices (including the apnea hypopnea index [AHI], micro-arousal index [MI], oxygen desaturation index [ODI]) and heart rate variability (HRV) parameters using Spearman’s correlation analysis. Moreover, we used multiple linear regression analyses to construct linear models for the AHI, MI, and ODI. In our analysis, the AHI was significantly associated with relative powers of very low frequency (VLF [%]) (r = 0.641, P = 0.001), relative powers of high frequency (HF [%]) (r = -0.586, P = 0.002), ratio between low frequency and high frequency powers (LF/HF) (r = 0.545, P = 0.049), normalized powers of low frequency (LF [n.u.]) (r = 0.506, P = 0.004), and normalized powers of high frequency (HF [n.u.]) (r = -0.506, P = 0.010) among patients with OSAHS. The MI was significantly related to standard deviation of RR intervals (SDNN) (r = 0.550, P = 0.031), VLF [%] (r = 0.626, P = 0.001), HF [%] (r = -0.632, P = 0.001), LF/HF (r = 0.591, P = 0.011), LF [n.u.] (r = 0.553, P = 0.004), HF [n.u.] (r = -0.553, P = 0.004), and absolute powers of very low frequency (VLF [abs]) (r = 0.525, P = 0.007) among patients with OSAHS. The ODI was significantly correlated with VLF [%] (r = 0.617, P = 0.001), HF [%] (r = -0.574, P = 0.003), LF [n.u.] (r = 0.510, P = 0.012), and HF [n.u.] (r = -0.510, P = 0.012) among patients with OSAHS. The linear models for the PSG diagnostic indices were AHI = -38.357+1.318VLF [%], MI = -13.389+11.297LF/HF+0.266SDNN, and ODI = -55.588+1.715VLF [%]. However, the PSG diagnostic indices were not related to the HRV parameters among healthy participants. Our analysis suggests that HRV parameters are powerful tools to screen for OSAHS patients in place of PSG monitoring.

## Introduction

Obstructive sleep apnea hypopnea syndrome (OSAHS) has a prevalence of 2% to 4% among middle-aged adults and is an independent risk factor for many systemic disorders [1]. Adverse consequences related to OSAHS include hypertension [2], stroke [3], coronary artery disease [4], congestive heart failure [5], arrhythmias [6], type 2 diabetes [7], insulin resistance [8], neurocognitive function [9], depression [10] and motor vehicle accidents [11], all of which can seriously affect patient quality of life and life expectancy. Moreover, the healthcare costs of OSAHS gradually increase as do those associated with other systematic disorders [12]. Therefore, it is important to identify OSAHS in its early stages. OSAHS includes numerous disordered events (apneas, hypopneas and micro-arousals) reaching to five events/hour in patients with the following symptoms: loud snoring, breathing interruptions, waking up holding their breath, daytime sleepiness, unrefreshing sleep and fatigue [13].

Full-night polysomnography (PSG) is recommended as a routine procedure in the diagnosis of OSAHS [14]. During PSG monitoring to assess OSAHS, the following physiological signals are acquired: electroencephalography (EEG), electromyography (EMG), electrooculogram (EOG), oxygen saturation, electrocardiogram (ECG), body movement, and nasal airflow. However, in-laboratory PSG has numerous limitations including a prolonged waiting time depending on the available local resources, the need for trained individuals who have the ability to monitor technical adequacy, the inconvenience of an overnight sleep study, and the high expense of the study. Physicians also must inspect the above recordings to diagnose and assess OSAHS offline. Researchers have actively sought an alternative and efficient examination to screen for OSAHS.

Heart rate variability (HRV) reflects the status of the autonomic nervous system (ANS) in patients with physiological and pathological conditions [15], providing a unique index to identify OSAHS. Numerous studies have demonstrated that the recurrence of the progressive-bradycardia/abrupt-tachycardia pattern observed in patients with OSAHS is likely the response of ANS to apnoeic events [16]. Much published research has investigated the HRV indices of patients with OSAHS. They have mostly focused on the relationship between HRV and the varying severity of OSAHS [17], and the effects of diverse treatment modalities on the HRV parameters of patients with OSAHS [18] (e.g., continuous positive airway pressure treatment, Mandibular repositioning splint treatment, and different forms of surgery). However, little research has explored the associations among the apnea hypopnea index (AHI), micro-arousal index (MI), oxygen desaturation index (ODI), and HRV parameters simultaneously.

Our study explored the associations between various PSG diagnostic indices (including the AHI, MI, and ODI) and HRV parameters; moreover, we constructed linear models to describe them. We also analyzed the changes in autonomic nervous activity among patients with OSAHS during nocturnal sleep. We hope that this study provides a theoretical basis for the use of HRV as a diagnostic index to replace PSG among patients with OSAHS.

## Materials and Methods

### Participants

We retrospectively studied 25 patients with normal sinus rhythm (23 men and two women) who had OSAHS-characteristic symptoms and 27 healthy participants (24 men and three women) without OSAHS at Xili People’s Hospital of Shenzhen. All participants were enrolled from November 2013 to November 2014. We measured their heights and weights to calculate the body mass indices (BMIs) for all patients. Nine, four, and twelve patients had mild, moderate and severe OSAHS, respectively, in our study according to the PSG diagnostic sleeping report that two experienced doctors independently created. The exclusion criteria for this study were as follows: potential cardiovascular disease, diabetes mellitus, pulmonary disease, thyroid disease, automatic nervous system disorders, other diseases causing daytime sleepiness (e.g., narcolepsy, Willis-Ekbom syndrome, periodic limb movement disorder, simple snoring and upper airway resistance syndrome), a clinical history of treatment for OSAHS or a history of medication affecting the HRV analysis results. The ethics committee of Xili People’s Hospital of Shenzhen approved our study. Written consent forms were obtained from all participants.

### Sleep studies

Each participant underwent eight-hour nocturnal monitoring using PSG in bed. We informed all participants not to ingest alcohol or caffeine, nap or engage in long or strenuous exercises on the day of monitoring. During the PSG monitoring, we collected physiological data including EEG, EMG, EOG, ECG, oxygen saturation, body movement, and nasal airflow. Apnea was defined as the absence of respiration for more than 10 seconds. Hypopnea was defined as the reduction of at least 50% ventilation resulting in a decrease in arterial saturation of 4% or more. OSAHS was defined as apnea or hypopnea occurring no fewer than five times per hour, lasting for at least 10 seconds. The AHI records the number of apnea-plus-hypopnea events per hour during sleep. The MI was calculated using the average value of the micro-arousals related to hourly respiratory events during sleep. The ODI assesses the average number of oxygen desaturation events per hour during sleep.

### ECG data acquisition and statistics

Raw ECG data were acquired using a PSG machine. This machine collects ECG data by lead II. The sampling rate was 512 Hz. Because 8-hour ECG recordings are too large to analyze, we divided these signals into 96 segments to more easily and accurately distinguish R-peaks. Every five minutes was counted as one segment. The average HRV parameters of all segments were calculated as the monitored HRV parameters. We used the freely available software package ARTiiFACT 2.0 (Psychonomic Society, Inc.) to detect R-peaks and extract inter-beat intervals. After using ARTiiFACT to detect the RR intervals, one experienced doctor marked the missing R-peaks and deselected incorrect R-peaks. Next, the software computed all of the basic HRV parameters, including the time and frequency domain parameters, using the HRV analysis module. The time domain parameters were MEANRR, MEDIANRR, MEANHR, SDNN, RMSSD, NN50, and pNN50. Frequency bands were divided into very low frequency (0.003 to 0.04 Hz), low frequency (0.04 to 0.15 Hz), and high frequency (0.15 to 0.4 Hz) categories. The frequency domain parameters were VLF [%], LF [%], HF [%], LF/HF, LF [n.u.], HF [n.u.], VLF [abs], LF [abs], and HF [abs]. Table 1 describes the time domain and frequency domain parameters.

**Table 1:** The time domain and frequency domain parameters.

Parameters	Description
MEANRR	Mean of RR intervals
MEDIANRR	Median RR intervals
MEANHR	Mean of heart rate
SDNN	Standard deviation of RR intervals
RMSSD	Square root of the mean squared differences between successive RR intervals
NN50	Number of successive RR interval pairs differing more than 50 ms
pNN50	NN50 count divided by the total number of RR intervals
VLF [%]	Relative powers of very low frequency
LF [%]	Relative powers of low frequency
HF [%]	Relative powers of high frequency
LF/HF	Ratio between low frequency and high frequency powers
LF [n.u.]	Normalized powers of low frequency
HF [n.u.]	Normalized powers of high frequency
VLF [abs]	Absolute powers of very low frequency
LF [abs]	Absolute powers of low frequency
HF [abs]	Absolute powers of high frequency

Data are expressed as the mean±SD. All data were analyzed using the statistical software SPSS 17.0 (SPSS Inc., Chicago, IL, USA). We used a two-tailed Spearman’s correlation analysis to explore the linear relationship between the OSAHS diagnostic indices and the HRV parameters. We used multiple linear regression analyses to construct linear models for AHI, MI, and ODI. These models were adjusted for baseline characteristics including age, gender, BMI, and neck circumference. We performed a power analysis for statistics. Power>0.75 was considered as reliable. P<0.05 was considered as significant.

## Results

The mean age, height, weight, BMI, and neck circumference of the OSAHS group were 36.96±11.09 (range = 4–53) years old, 164.48±12.53 (range = 115–178) cm, 79.88±15.23 (range = 21.5–105) kg, 29.01±3.35 (range = 16.3–33.1) kg/m^2^, and 41.18±3.46 (range = 28.1–45.3) cm, respectively. These values were 38.85±12.14 (range = 6–55) years old, 166.12±13.50 (range = 135–179) cm, 75.88±13.23 (range = 25–103) kg, 27.01±2.44 (range = 15.9–32.8) kg/m^2^, and 37.18±4.53 (range = 30.3–46.0) cm for the control group, respectively.

The means±SDs of MEANRR, MEDIANRR, MEANHR, SDNN, RMSSD, NN50 and pNN50 of the time domain variables are presented for all patients in Table 2. Table 2 lists the correlation coefficients between the time domain parameters and the AHI, MI, and ODI for the OSAHS group. Similarly, Table 3 shows the means±SDs of the time domain parameters and their relationships with regard to the control group. Our analysis shows that the correlations of the OSAHS diagnostic indices and the time domain variables were not significant except for the positive correlation between the MI and SDNN (r = 0.432, P = 0.031) among patients with OSAHS. The power values for r-value are greater than 0.75. The correlations between the PSG diagnostic indices and the time domain parameters were not significant with regard to the control group.

**Table 2:** Correlations between the time domain parameters and AHI, MI, and ODI with regard to patients with OSAHS.

Parameters	Mean±SD	AHI		MI		ODI	
		r	P	r	P	r	P
MEANRR	841.95±111.78	-0.205	0.327	-0.044	0.835	-0.186	0.373
MEDIANRR	844.99±113.84	-0.180	0.389	-0.017	0.936	-0.162	0.438
MEANHR	72.80±9.33	0.212	0.310	0.052	0.807	0.192	0.359
SDNN	72.07±24.77	0.171	0.414	0.550	0.031*	0.246	0.236
RMSSD	52.65±27.67	-0.126	0.548	0.079	0.707	-0.058	0.781
NN50	66.94±44.29	-0.105	0.616	0.171	0.414	0.028	0.895
pNN50	19.91±13.69	-0.119	0.570	0.155	0.461	0.009	0.967

* P<0.05.

**Table 3:** Correlations between the time domain parameters and the AHI, MI, and ODI with regard to controls.

Parameters	Mean±SD	AHI		MI		ODI	
		r	P	r	P	r	P
MEANRR	801.32±130.09	-0.198	0.341	-0.038	0.764	-0.177	0.324
MEDIANRR	800.21±160.24	-0.201	0.353	-0.026	0.876	-0.142	0.415
MEANHR	70.05±10.97	0.209	0.334	0.044	0.754	0.173	0.301
SDNN	65.01±26.87	0.167	0.385	0.654	0.047	0.241	0.316
RMSSD	48.52±30.55	-0.116	0.614	0.062	0.866	-0.046	0.569
NN50	60.04±35.89	-0.108	0.578	0.211	0.531	0.035	0.743
pNN50	16.11±11.75	-0.114	0.611	0.162	0.489	0.013	0.795

**Fig 1:**
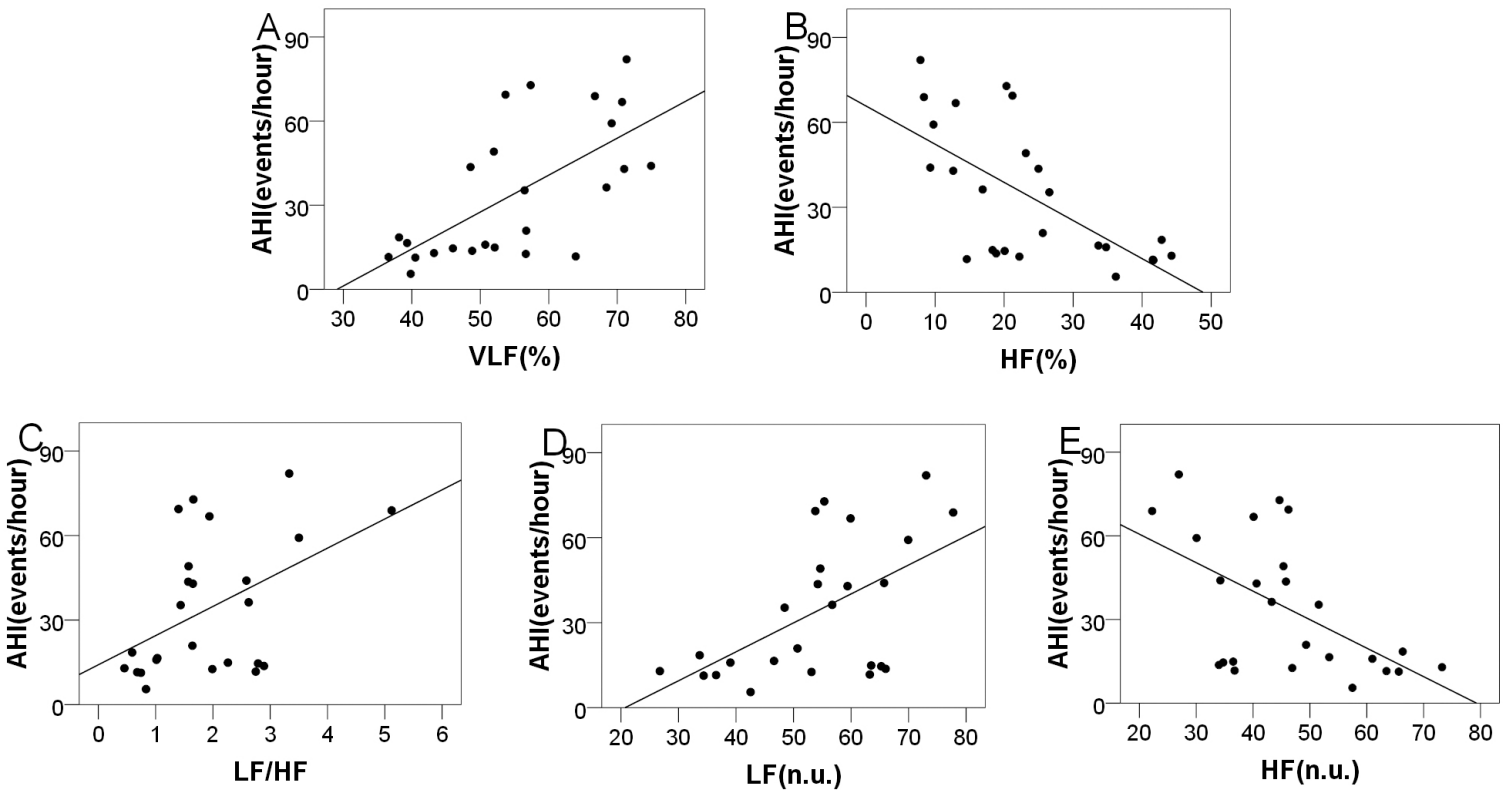
The relationship between the frequency domain parameters and the AHI with regard to the OSAHS group. (A) Scatterplot of VLF [%] with AHI; (B) Scatterplot of HF [%] with AHI; (C) Scatterplot of LF/HF with AHI; (D) Scatterplot of LF [n.u.] with AHI; (E) Scatterplot of HF [n.u.] with AHI.

The AHI, MI and ODI were significantly correlated with different frequency domain variables with regard to the OSAHS group. The mean AHIs were 34.03±24.09 (range = 5.5–82.0) and 2.11±2.08 (range = 2.1–4.9) for the OSAHS and control groups, respectively. As Table 4 shows, the AHI was significantly and positively correlated with VLF [%] (r = 0.641, P = 0.001), LF/HF (r = 0.545, P = 0.049), and LF [n.u.] (r = 0.557, P = 0.004) but negatively correlated with HF [%] (r = -0.586, P = 0.002) and HF [n.u.] (r = -0.506, P = 0.010) with regard to the OSAHS group. The power values for all the r-value are greater than 0.75. However, the relationship between the AHI and the frequency domain indices was not significant regarding the control group. The scatterplots showing the relationship between these frequency domain parameters and the AHI are shown in Fig 1.

**Table 4:** The correlations between the frequency domain parameters and the AHI.

Parameters	AHI (OSAHS group)		AHI (control group)	
	r	P	r	P
VLF [%]	0.641	0.001**	0.532	0.065
LF [%]	-0.050	0.812	-0.033	0.789
HF [%]	-0.586	0.002**	-0.425	0.059
LF/HF	0.545	0.049*	0.356	0.060
LF [n.u.]	0.506	0.010**	0.465	0.056
HF [n.u.]	-0.506	0.010**	-0.465	0.056
VLF[abs]	0.283	0.170	0.274	0.210
LF [abs]	0.082	0.696	0.069	0.834
HF [abs]	-0.253	0.222	-0.195	0.332

* P<0.05;

** P<0.01.

**Fig 2:**
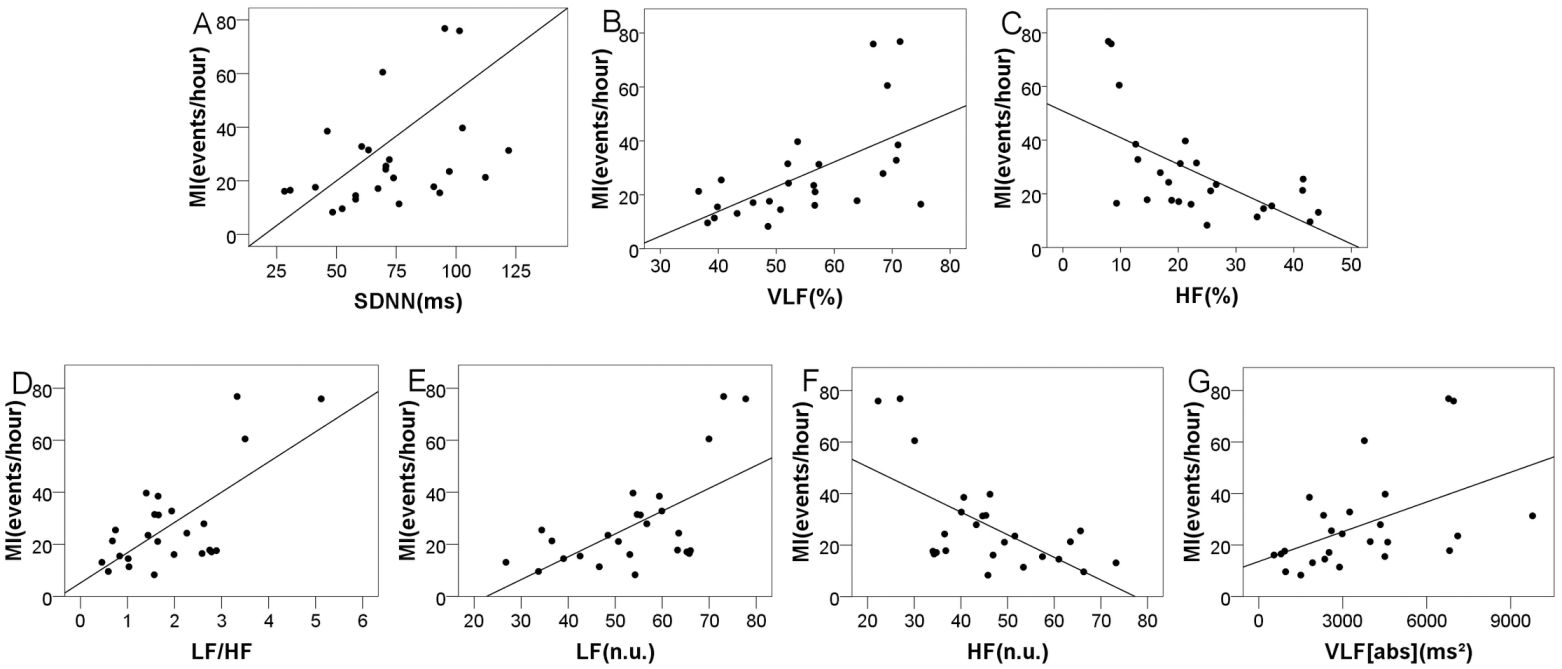
The relationship between the HRV domain parameters and the MI regarding the OSAHS group. (A) Scatterplot of SDNN with MI; (B) Scatterplot of VLF [%] with MI; (C) Scatterplot of HF [%] with MI; (D) Scatterplot of LF/HF with MI; (E) Scatterplot of LF [n.u.] with MI; (F) Scatterplot of HF [n.u.] with MI; (G) Scatterplot of VLF [abs] with MI.

The mean MIs were 27.52±18.62 (range = 8.3–60.5) and 6.10±5.24 (range = 4.2–13.0) for the OSAHS group and control group, respectively. Table 5 shows that the MI positively correlated with VLF [%] (r = 0.626, P = 0.001), LF/HF (r = 0.591, P = 0.011), LF [n.u.] (r = 0.553, P = 0.004), and VLF [abs] (r = 0.525, P = 0.007) but negatively correlated with HF [%] (r = -0.632, P = 0.001) and HF [n.u.] (r = -0.553, P = 0.004) with regard to the OSAHS group. The power values for all the r-value are greater than 0.75. However, the MI was not significantly related to the frequency domain indices regarding the control group. The linear relationship between the HRV parameters and the MI is depicted in Fig 2.

**Table 5:** The correlations between the frequency domain parameters and the MI.

Parameters	MI (OSAHS group)		MI (control group)	
	r	P	r	P
VLF [%]	0.626	0.001**	0.433	0.058
LF [%]	-0.022	0.919	-0.029	0.942
HF [%]	-0.632	0.001**	-0.363	0.064
LF/HF	0.591	0.011*	0.254	0.089
LF [n.u.]	0.553	0.004**	0.233	0.672
HF [n.u.]	-0.553	0.004**	-0.233	0.672
VLF[abs]	0.525	0.007**	0.346	0.535
LF [abs]	0.374	0.066	0.179	0.089
HF [abs]	-0.085	0.688	-0.054	0.599

* P<0.05;

** P<0.01.

**Fig 3:**
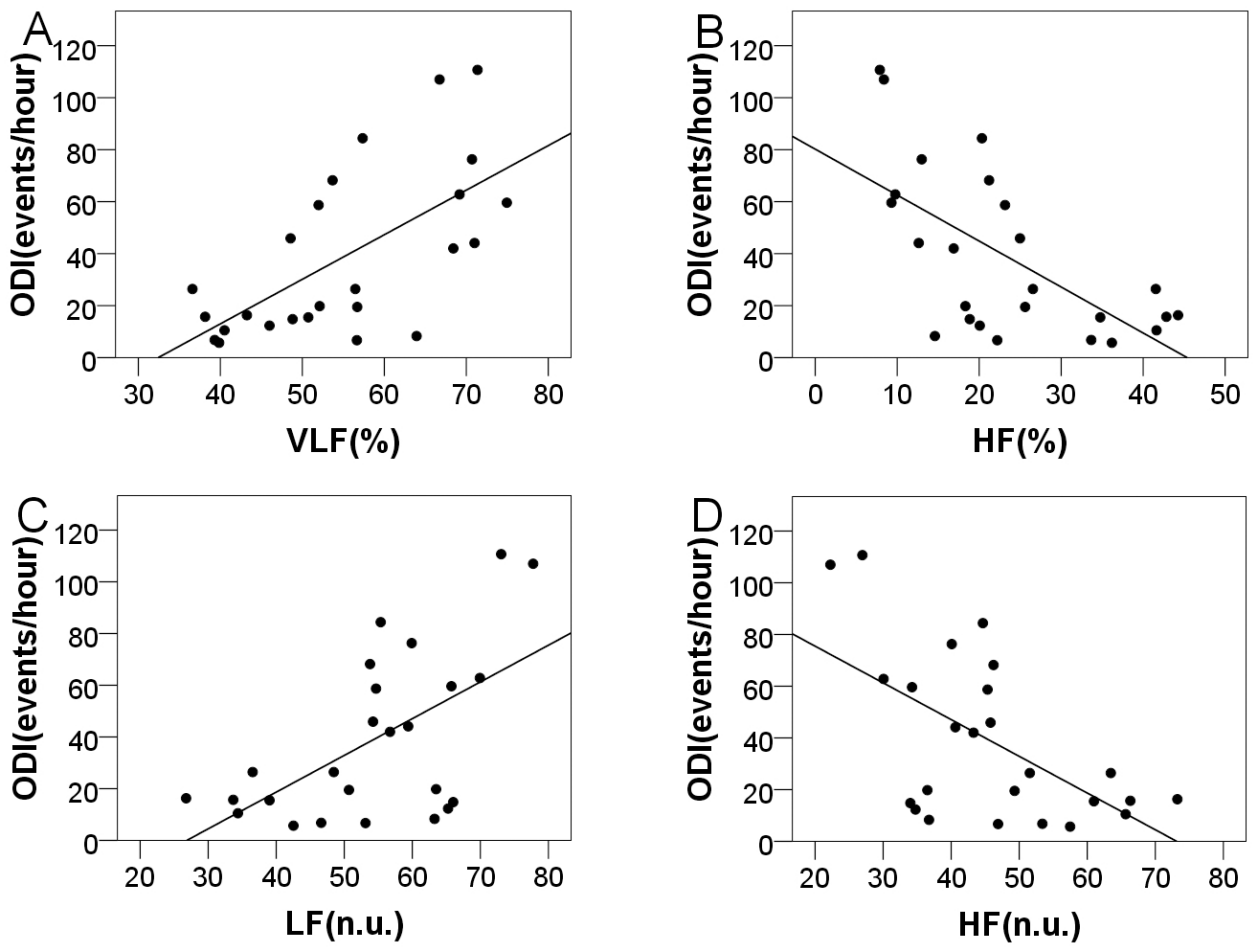
The relationship between the frequency domain parameters and the ODI with regard to the OSAHS group. (A) Scatterplot of VLF [%] with ODI; (B) Scatterplot of HF [%] with ODI; (C) Scatterplot of LF [n.u.] with ODI; (D) Scatterplot of HF [n.u.] with ODI.

The mean ODIs were 38.58±31.92 (range = 5.7–110.7) and 1.40±2.01 (range = 1.2–4.2) with regard to the OSAHS and control groups, respectively. Table 6 shows the correlation coefficients between the ODI and the frequency domain parameters for these two groups. The ODI was positively correlated with VLF [%] (r = 0.617, P = 0.001) and LF [n.u.] (r = 0.510, P = 0.012) but negatively correlated with HF [%] (r = -0.574, P = 0.003) and HF [n.u.] (r = -0.510, P = 0.012) among patients with OSAHS. The power values for all the r-value are greater than 0.75. No significant correlations were observed with regard to the frequency domain indices for the control group. Fig 3 shows the linear relationships between the frequency domain parameters and the ODI.

**Table 6:** The correlation between the frequency domain parameters and ODI.

Parameters	ODI (OSAHS group)		ODI(control group)	
	r	P	r	P
VLF [%]	0.617	0.001**	0.412	0.056
LF [%]	-0.118	0.575	-0.105	0.622
HF [%]	-0.574	0.003**	-0.337	0.089
LF/HF	0.358	0.079	0.229	0.108
LF [n.u.]	0.510	0.012*	0.421	0.063
HF [n.u.]	-0.510	0.012*	-0.421	0.063
VLF[abs]	0.328	0.110	0.227	0.291
LF [abs]	0.160	0.445	0.131	0.611
HF [abs]	-0.185	0.375	-0.106	0.452

* P<0.05;

** P<0.01.

Based on Spearman’s correlation analysis between the PSG diagnostic indices and the HRV parameters for the OSAHS group, multiple regression analyses were performed using stepwise selection to construct linear models. Table 7 depicts three regression models for the AHI, MI, and ODI. In model 1, AHI was the dependent variable, and VLF [%] was the independent variable. The model was AHI = -38.357+1.318VLF [%]. A similar result was found with regard to the ODI, where VLF [%] was the independent factor, and the ODI was the dependent factor in model 2. The model was ODI = -55.588+1.715VLF [%]. LF/HF and SDNN served as independent variables, and the MI was the dependent variable in model 3. The model was MI = -13.389+11.297LF/HF + 0.266SDNN. An ANOVA indicated that all of the models were significant. Table 7 depicts the multiple regression analyses for the AHI, MI, and ODI.

**Table 7:** Multiple regression analyses for the AHI, MI, and ODI with regard to the OSAHS group.

Model	Variable	B	β	R^2^	R^2^_adj_	ANOVA	
						F	P value
1	AHI			0.427	0.402	17.149	0.000
	VLF [%]	1.318	0.654**				
	Constant	-38.357					
2	MI			0.591	0.554	15.914	0
	LF/HF	11.297	0.664**				
	SDNN	0.266	0.354*				
	Constant	-13.389					
3	ODI			0.412	0.386	16.107	0.001
	VLF [%]	1.715	0.642**				
	Constant	-55.588					

Dependent variables: AHI for model 1, MI for model 2, and ODI for model 3; Independent variables: VLF [%] for model 1, LF/HF and SDNN for model 2, and VLF [%] for model 3; B: unstandardized coefficient; β: standardized coefficient; R^2^_adj_: adjusted R^2^;

*P<0.05;

**P<0.01.

## Discussion

OSAHS is receiving increased attention because of growing concern regarding its effect on quality of life; thus, its diagnosis is particularly important. We attempted to make this complicated, traditional examination easier by analyzing the correlation between PSG indices and HRV parameters. PSG reflects respiratory and oxygenation patterns [19]. Therefore, the AHI, MI, and ODI are widely accepted as indices to diagnose and assess OSAHS. Previously, Guilleminault et al. proposed that sleep apnea syndrome elicited changes in heart rate rhythm that were mediated by the automatic nervous system [20]. HRV is a noninvasive, simple, and affordable method that indicates not only cardiovascular disease but also the conditions of patients with other diseases [17, 21, 22].

Time domain parameters are frequently used to investigate OSAHS. Roche et al. found that the SDNN index and RMSSD remained significant predictors of OSAHS [23]. In our study, we found that the MI was significantly and positively correlated with SDNN. Similarly, Kim et al. concluded that SDNN was higher in patients with OSAS than controls [17, 24]. Because SDNN only quantifies the total variability of heart rate during recording, it provides limited information when a sympathovagal imbalance is detected. Time domain parameters are simple measurements of the HRV present in an ECG. All time domain indices are influenced by variations in both sympathetic and parasympathetic activity, which make them non-specific measures of sympathovagal balance [25]. Thus, the role that time domain parameters play in quantifying specific changes in sympathetic or parasympathetic activity is limited. We recognize the association between the MI and SDNN among participants with OSAHS, but SDNN does not determine the value of this index.

Currently, spectral analyses of HRV are a frequently used to assess the autonomic modulation of heart rate. Spectral analyses provide precise information on sympathetic and parasympathetic function [26]. In addition, spectral analyses of HRV have high specificity and sensitivity. Moreover, they are more reproducible than time domain methods over short-term investigation of HRV [27]. Park found that the AHI was significantly correlated with VLF power, LF power and the LF/HF ratio [17]. Likewise, Kim et al. analyzed the correlations between the AHI and HRV parameters and found that the correlation with the LF/HF ratio was significant among patients with OSAHS [24]. The present study found complementary findings. We found that the AHI was significantly and positively correlated with VLF [%], HF [%], LF/HF, LF [n.u.], and HF [n.u.]. In addition, the MI was significantly associated with VLF [%], HF [%], LF/HF, LF [n.u.], HF [n.u.], and VLF [abs]. However, the ODI was associated with VLF [%], HF [%], LF [n.u.], and HF [n.u.]. The physiological mechanisms underlying HRV analyses are only partially understood. Parasympathetic activation slows heart rate. The synaptic release of acetylcholine mediates this phenomenon, which is characterized by a brief incubation period and a high turnover rate [25]. The biological mechanism of rapid response enables the parasympathetic nervous system to mediate cardiac pumping on a beat-to-beat basis. In contrast, sympathetic activation increases heart rate and conduction system velocity, together with an enhancement in contractility, mediated by the synaptic release of noradrenaline [25]. Absorption and metabolism are relatively slow. Therefore, the changes in cardiovascular function mediated by the alterations in sympathetic activation have a slower time course. Because of the differences in neurotransmitters, the two branches of the ANS tend to manipulate different frequencies of heart rate. The predominant changes in the sympathetic or parasympathetic systems can be identified and quantified [28–30].

A previous study showed that cyclical changes in heart rate are associated with respiration [31]. Respiration related to variation occurring at a high frequency might be abolished by vagal blockade [32]. The above results suggest that the parasympathetic system mediated the high frequency. Some authors have proposed that LF power is significantly affected by the sympathetic nervous system [32, 33]. However, recent research demonstrated that the parasympathetic nervous system also mediated low frequency, suggesting that a vagal component also exists with regard to this cyclic activity [25]. Because of the dual mechanisms of mediation, LF powers cannot be regarded as a quantitative index of sympathetic activity. As for VLF powers, no agreement explains the meaning of the VLF component. In general, VLF is considered as correlated with changes in parasympathetic activation [34], the activity of the renin-angiotensin system [35], peripheral chemoreceptor activity [36] [37] and thermoregulatory mechanisms [38].

Parasympathetic activity predominated in supine and quiet positions, whereas the predominance of sympathetic activity was lesser [25]. Once partial or complete closure of the upper airway occurred repeatedly, apnea, hypopnea, or both arrived in quick succession [39]. Therefore, hypoxemia and awakening occurred in patients with OSAHS. The absence of air exchange and relative hypoxemia might act on central chemoreceptors [34]. As a result, sympathetic predominance replaced vagal predominance. Hypoxemia augments sympathetic activity and increases the release of catecholamines [40, 41]. The augmentation of HF powers is related to increased parasympathetic activity and synchronized with inspiratory efforts [34, 42]. In patients with OSAHS, repeated episodes of hypoxia and micro-arousal might result in less parasympathetic activity and more sympathetic activity. The decrease of parasympathetic activity represents a decline in HF power. In summary, the values of the AHI, MI, and ODI increase as HF power decrease. Likewise, the increase of sympathetic activity implies an increase in LF power. Thus, the values of the AHI, MI, and ODI increase as LF power increases. The present study found that the AHI, MI and ODI can be used to diagnose and assess OSAHS because of their negative correlations with HF [%] and HF [n.u.] as well as their positive correlation with LF [n.u.]. The LF/HF ratio was higher with increased LF and decreased HF. This finding explains why the AHI and MI increase with increases in the LF/HF ratio. The explanation for VLF is the most controversial.

Shiomi et al. found augmentation in VLF and detected a VLF peak in patients with OSAHS [43]. In that same study, the authors suggested that VLF increased synchronously with respiratory arrest or hypoxemia [43]. These results are consistent with our findings showing that AHI, MI, and ODI were positively related to VLF [%] and that the MI was positively related to VLF [abs]. The present study tended to support the view that VLF is related to peripheral chemoreceptor activity and the stimulation of the renin-angiotensin system, causing by hypoxemia and hypercapnia. In addition, the AHI and ODI models were AHI = -38.357+1.318VLF [%] and ODI = -55.588+1.715VLF [%], respectively. These models suggest that researchers should pay more attention to the VLF band of HRV. No matter what the units was, frequency domain parameters could reflect VLF, LF and HF practically, but it was analyzed that the normalized frequency domain parameters was better measures of ANS than absolute numbers [44].

In general, we found that the AHI, MI, and ODI showed significant linear relationships with certain frequency domain parameters for the OSAHS group. We also built linear models for the AHI, MI, and ODI. The models were AHI = -38.357+1.318VLF [%], MI = -13.389+11.297LF/HF + 0.266SDNN, and ODI = -55.588+1.715VLF [%]. We found many single correlations between the PSG diagnostic indices and the HRV parameters among patients with OSAHS. We should use these three models to accurately estimate the AHI, MI, and ODI. Therefore, we speculate that VLF [%], LF/HF, and SDNN are meaningful HRV parameters for clinical use. The data obtained using non-invasive methods might be more accurate than those using invasive methods because of changes in the ANS caused by the latter [45]. Based on the above significant correlations and the simplicity and non-invasiveness of the technique, HRV parameters should be applied to diagnose and assess OSAHS.

Finally, the present study has several limitations. First, it is obvious that PSG diagnostic indices associated with more than one HRV parameters in our study. It is a major limitation for us not to make multiple comparisons. In the subsequent research, we will improve the multiple comparisons. Second, the size of the sample was relatively small. Third, we performed a single institutional study. Forth, no subgroups were created based on severity. We are looking forward to performing large-sample and multi-center research. At last, we did not account for the changes in sleep stage that might also influence the frequency domain parameters [34]. Changes in the relationship between the PSG diagnostic indices and the HRV parameters are expected across different sleep stages. In addition, further research is required to explore the specific meaning of the VLF powers.

## Conclusions

OSAHS is a complex medical condition that results in high morbidity and mortality rates across both developed and developing regions. The current study found significant associations between PSG diagnostic indices and HRV parameters as well as constructed linear models for the AHI, MI, and ODI with regard to OSAHS participants. The recurrence of apnea, hypopnea, and micro-arousal could result in the changes in the ANSs of OSAHS participants. HRV indicators represent the sympathetic, parasympathetic, and sympathetic-parasympathetic regulation of heart rate. PSG examination is relatively expensive and time-consuming. However, ECG examination is efficient and cheap. The strong significant correlation and our established models show that HRV examination can replace cumbersome sleep monitoring as a powerful tool to diagnose and assess the status of OSAHS.

## Supporting Information

S1 Dataset Raw data of heart rate variability underlying the findings in our study in the manuscript. (XLSX)
